# Hospital Finance

**Published:** 1922-12

**Authors:** 


### HOSPITAL FINANCE.

### THE LATEST CONTRIBUTORY SCHEME.

THE four voluntary hospitals of Sheffield—the Royal Hospital, Royal Infirmary, Jessop Hospital for Women and Children's Hospital have joined forces (with the Edgar Allen Institute in the combination) to form a Hospitals Contributing Scheme. The project does not greatly differ from other schemes elsewhere, but it seeks incidentally to make uniform all Sheffield systems of workmen's contributions, the hospitals pooling the results and giving in return " benefits " to the contributors.

An offer is made that employees of Sheffield firms shall have hospital treatment when needed for themselves or dependants, free of any charge for maintenance, in return for a contribution of a penny in each completed pound of earnings. Employers are asked to add not less than one-third to the total of their employees' contributions. Non-contributors suitable for hospital treatment will, of course, as hitherto be received, but they and their dependants will be required to pay adequately towards cost of maintenance. The Sheffield Joint Hospitals' Council, St. Peter's Close, Hartshead, Sheffield, of which Mr. S. R. Lamb is the Secretary, administers the scheme. Sheffield being one of the most important industrial centres has been made a separate region by both the Voluntary Hospitals Commission and the British Hospitals Association. In the attractive booklet describing the scheme, issued by the Council, it is stated that arrangements have been made with the Inland Revenue authorities that all annual subscriptions to hospitals and to the Sheffield Hospitals' Council may be included in their trading accounts for relief from income tax.

### THE NEW HOSPITAL MOVEMENT.

LAST year a movement started at the Middlesex Hospital to encourage workers and residents in the locality to support their local hospitals, known as the Local Appeal Fund of the St. Marylebone Associated Hospitals. The movement has thriven, and begun a new season's activity with a series of social and scientific gatherings on the Fund's behalf. St. Marylebone has given a good lead in this matter, and the financial results will probably ensure imitation in other Boroughs. Major J. R. Ainsworth Davis, the organiser of the local appeal, has already explained in " The Times " that the Borough contains nine hospitals, whose co-operation is securing the support of the big and small employers and their staffs, after which the private residents will be interested. Major Davis believes that the support of the hospitals mainly depends upon the staffs of the firms in each London area, a refreshing conviction that has taken many years to implant itself in London. He has therefore encouraged staff councils among the employees, and committees to create them have been appointed in each of the nine wards of Marylebone, all related to the headquarters of the movement, at 66 Goodge Street. An important adjunct will be the proposed Hospital Guild about to be formed to bring the staffs of the different firms together not merely as co-workers, but in a human and social way. Hence the season's programme of social and scientific meetings. The subjects of these meetings will include a demonstration of the work accomplished by the nine hospitals in St. Marylebone. At the first meeting, for example, Dr. J. H. D. Webster gave a demonstration of X-rays, Professor James McIntosh, of the use of the microscope in research, and Mr. W. H. S. Cheavin, of the microscope's value in the detection of forgeries. Nor did these exhaust the list, and the evening ended with an organ recital by Mr. H. H. Stubbs in the hospital chapel. Already, we understand, the Borough of Westminster is following suit, and there seems no reason why the " movement " should not become general. It will educate, without their knowing it, the members who attend the gatherings, and not only these but the hospitals concerned in the advantages of combination. Since so many patients come to London hospitals from outside, it would be too much to desire that each Borough should bear the whole burden of hospital maintenance in its area. But each should wish to bear a fair share, and the " new movement " provides the local firms and workers with an excellent and agreeable means of so doing. We have been fortunate in securing from Major Davis a detailed statement of the principles behind, and the methods shown to be suited to, the new policy. Since these are of general application, they should encourage other groups of hospitals to fall into line. Major Davis' article appears on page 37.

### HOSPITAL TRADING.

IN our last issue we reviewed the address of Mr. H. J. Waring on " The Future of Hospitals," in which he advocated the building, in connection with voluntary hospitals, of departments for paying patients which could be made a source of profit—a most useful suggestion in these days when the difficulty of raising funds is so great. In the " Lancet," Sir James P. J. Michelli, secretary to the Seamen's Hospital, has pointed out several extensions of the proposal, which we commend to hospital governors. The pharmacy unit can be enlarged and drugs sold to the general public. The pathological department can be made a paying concern by extending its scope and making vaccines and examining specimens for public bodies, general practitioners and others. The massage and X-ray departments can be extended in like manner. All can be run at a profit in the interests of the hospital, but beyond this there is the advantage that the departments will be larger and cover a wider scope, more persons will be employed, and more liberal remuneration paid to the professional and subordinate *personnel*, while opportunities for research will be extended. Thus each of these departments, instead of being an expense, will help to finance the hospital. The advance of medical science and the cost entailed thereby has done much to paralyse the voluntary system, and here is the way out.

